# The healthful plant‐based diet index as a tool for obesity prevention—The healthy lifestyle community program cohort 3 study

**DOI:** 10.1002/osp4.649

**Published:** 2022-12-25

**Authors:** Christian Koeder, Dima Alzughayyar, Corinna Anand, Ragna‐Marie Kranz, Sarah Husain, Nora Schoch, Andreas Hahn, Heike Englert

**Affiliations:** ^1^ Institute of Food Science and Human Nutrition Leibniz University Hannover Hannover Germany; ^2^ Department of Nutrition University of Applied Sciences Münster Münster Germany

**Keywords:** cardiovascular disease, healthy diet, lifestyle medicine, obesity prevention, plant‐based diet, weight loss

## Abstract

**Background:**

World‐wide the prevalence of obesity is high, and promoting a shift toward more healthful and more plant‐based dietary patterns appears to be one promising strategy to address this issue. A dietary score to assess adherence to a healthy plant‐based diet is the healthful plant‐based diet index. While there is evidence from cohort studies that an increased healthful plant‐based diet index is associated with improved risk markers, evidence from intervention studies is still lacking.

**Methods:**

A lifestyle intervention was conducted with mostly middle‐aged and elderly participants from the general population (*n* = 115). The intervention consisted of a 16‐month lifestyle program focusing on a healthy plant‐based diet, physical activity, stress management, and community support.

**Results:**

After 10 weeks, significant improvements were seen in dietary quality, body weight, body mass index, waist circumference, total cholesterol, measured and calculated low‐density lipoprotein (LDL) cholesterol, oxidized LDL particles, non‐high‐density lipoprotein cholesterol, remnant cholesterol, glucose, insulin, blood pressure, and pulse pressure. After 16 months, significant decreases were seen in body weight (−1.8 kg), body mass index (−0.6 kg/m^2^), and measured LDL cholesterol (−12 mg/dl). Increases in the healthful plant‐based diet index were associated with risk marker improvements.

**Conclusions:**

The recommendation of moving toward a plant‐based diet appears acceptable and actionable and may improve body weight. The healthful plant‐based diet index can be a useful parameter for intervention studies.

## Graphical abstract

The Healthy Lifestyle Community Program (cohort 3) achieved a significant reduction in body weight (−1.8 kg) in a community‐based sample of mostly clinically healthy participants. This weight loss was sustained at the end of the study (16 months). Adherence to a healthy plant‐based diet correlated with improved risk markers.

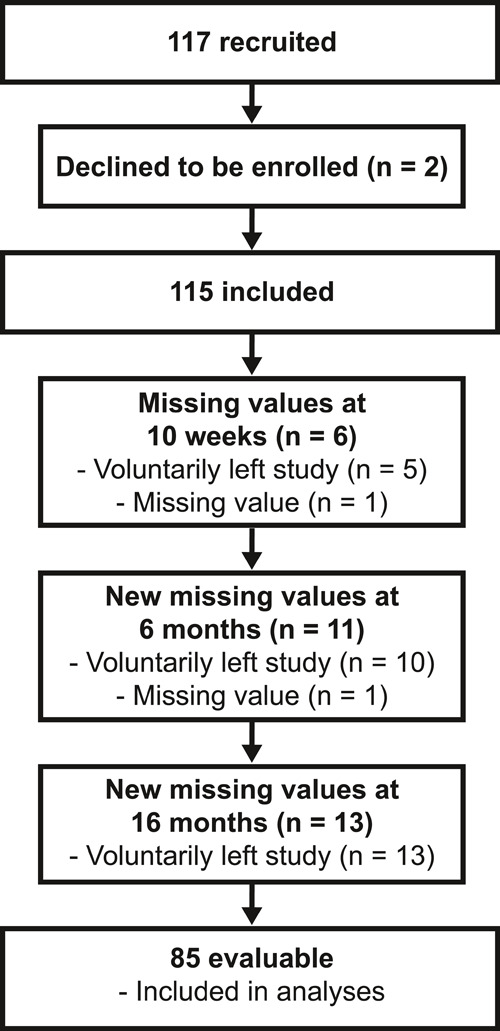

## 1 INTRODUCTION

Obesity is associated with an increased risk of chronic disease, including diabetes,^1^ and cardiovascular disease (CVD), as well as with increased all‐cause mortality.^2^ The adverse effects of obesity on health appear to be partly (but not entirely) mediated by its adverse effects on established risk factors.^2^ Diet can be considered one of the most critical factors for weight management,^3^ and current evidence increasingly indicates that moving from a hypercaloric, typical Western diet toward a healthier, fiber‐rich, and predominantly plant‐based dietary pattern could, at the population level, favorably affect body weight,^4^ cholesterol levels, blood pressure^5^ as well as markers of glycemic control^6^ and inflammation.^7^ Adopting such a dietary pattern would also be in line with current guidelines for CVD prevention.^8^, ^9^ Uncertainty remains, however, how this knowledge can be applied, as a public health measure, to reach a wider audience of citizens, that is, how the public can be encouraged and enabled to change their food habits toward a well‐planned, healthier, and more plant‐based diet.^10^

The plant‐based diet index (PDI), healthful PDI (hPDI), and unhealthful PDI (uPDI) have been used in a considerable number of studies since these indices were first published in 2016.^11^ The PDI is a measure of adherence to a plant‐based diet in general, while hPDI and uPDI are measures of adherence to a healthy and unhealthy plant‐based diet, respectively.^12^ To date, these plant‐based diet scores have been used mostly in large cohort studies.^13^, ^14^, ^15^ Results from cohort studies support the theory that higher intakes of healthy plant‐based foods and concomitant lower intakes of unhealthy plant‐based foods (such as added sugars and refined grains) and of animal‐source foods are associated with a reduced CVD risk.^13^ However, evidence is lacking on whether associations between changes in these easy‐to‐use indices, which are based on food groups, and changes in body weight and other CVD markers can be shown in intervention studies.^16^ To date, no intervention trials appear to have tested such associations. However, a secondary analysis of the PREVIEW trial (a large, 3‐year, international, multicenter randomized controlled trial) has assessed the association of PDI change with CVD marker changes and found that an increase in PDI was associated with improved body weight maintenance.^16^ This indicates that the use of PDI (as well as hPDI and uPDI) may be feasible for intervention studies, particularly those including dietary recommendations which are similar to what the hPDI score indicates and measures, that is, to consume fewer animal‐source foods and fewer unhealthy plant foods and instead more healthy plant foods.^10^, ^12^

At present, however, it is uncertain whether a lifestyle program including the recommendation to adopt a healthy plant‐based diet and other healthy lifestyle practices is acceptable for rural Western populations (who often follow a traditional diet high in different kinds of meat and processed meats as well as eggs, cheese, cream, butter, lard, and potatoes^17^), and it is also uncertain whether such programs would result in improved risk markers. In addition, evidence is lacking on whether the plant‐based diet indices PDI, hPDI, and uPDI are a useful tool to illustrate the association between improved dietary quality and improved risk markers in the context of intervention studies.

Measures to prevent chronic diseases are often taken relatively late in life and, thus, the window of greatest opportunity (i.e., early prevention) is frequently missed.^18^, ^19^ For example, although it is known that atherosclerosis development depends on exposure to risk factors over the course of one's lifetime and that early signs of atherosclerosis can already be detected in childhood,^20^ traditional risk scores categorize a large portion of the population as having a low to intermediate risk of CVD events.^18^ Consequently, the general population is often under the impression that preventative measures against CVD need only be taken later in life and when their CVD risk is categorized as high.^18^, ^19^

Against this background, the objective of the present study was to test whether the community‐based lifestyle intervention “Healthy Lifestyle Community Program cohort 3” (HLCP‐3) can improve body weight and other CVD risk markers in a heterogenous sample of middle‐aged and elderly participants (most of whom were clinically healthy). Furthermore, the present study had the objective to assess whether changes in the plant‐based diet indices (PDI, hPDI, and uPDI) would correlate with risk marker changes. The study's hypothesis was that the program (HLCP‐3) would be effective at improving the assessed CVD risk markers.

## 2 MATERIALS AND METHODS

### 2.1 Study design

An uncontrolled lifestyle intervention was conducted between March 2019 and July 2020. Assessments were made at baseline (March 2019), 10 weeks (June 2019), 6 months (October 2019), and 16 months (July 2020). The 16‐month measurement time point had originally been planned to take place after 12 months but was delayed due to the COVID‐19 pandemic. The time points which had originally been planned for 18 and 24 months were canceled due to the pandemic. Participants were recruited from the general population in rural northwest Germany.

### 2.2 Participants

**FIGURE 1:**
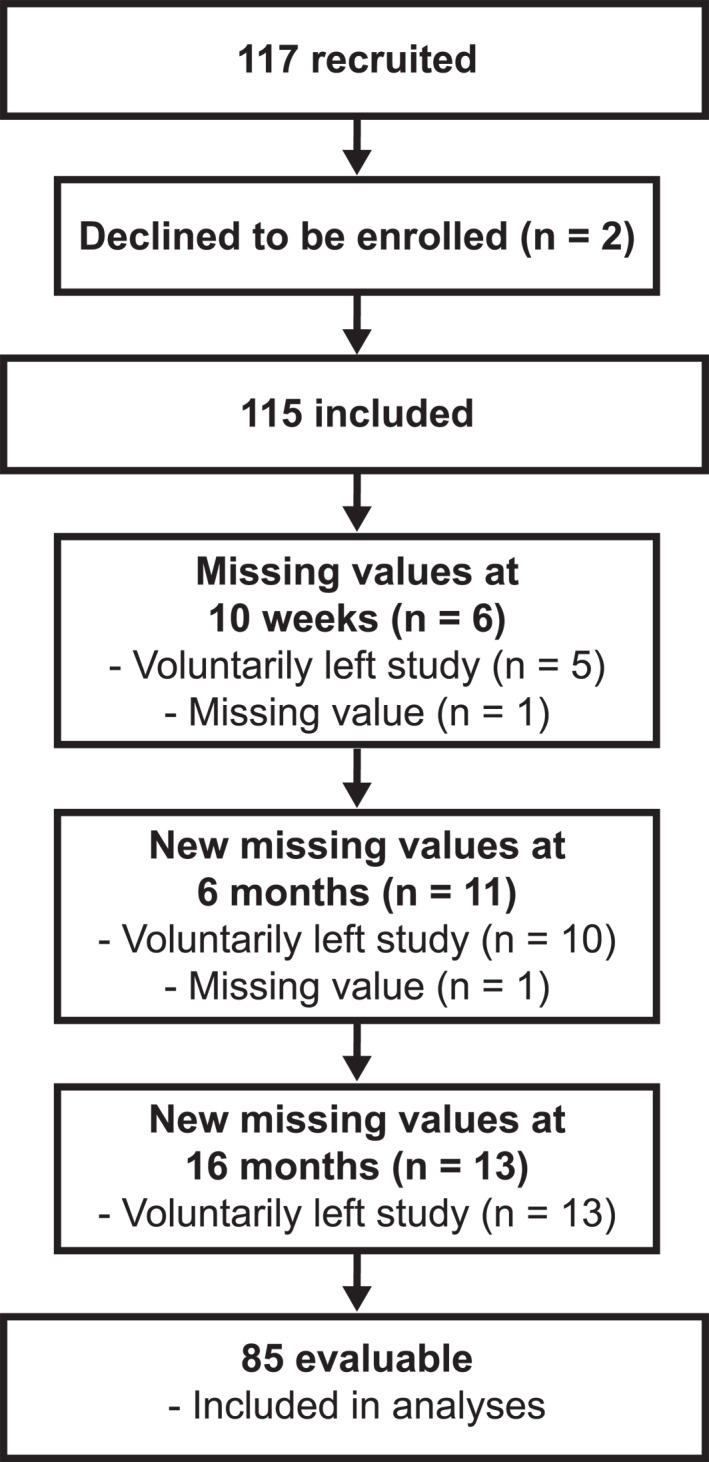
Flow chart of participants through the study.

Participants were mostly middle‐aged and elderly. The only inclusion criteria were the physical and mental ability to take part in the study (self‐reported) and to be ≥18 years of age. For the intervention, a total of 117 participants were recruited (Figure 1).

### 2.3 Lifestyle intervention

The lifestyle intervention HLCP‐3 was similar to the “Healthy Lifestyle Community Program cohort 2” (HLCP‐2) intervention, which has been described previously.^10^, ^21^ It consisted of an intensive phase (15 seminars, plus 8 workshops, in 10 weeks) and a less intensive alumni phase (for the remainder of the study; monthly seminars). Healthy lifestyle recommendations were given regarding diet, physical activity, stress management, and community support, with the strongest emphasis on diet. Dietary recommendations were to move from a typical German dietary pattern toward a more plant‐based diet, increasing the intake of fruit, vegetables, whole grains, legumes, nuts, seeds, cold‐pressed oils (such as olive oil, canola oil, flaxseed oil, and spices) as well as decreasing the intake of animal‐source foods (particularly meat but also eggs and high‐fat dairy products) and of unhealthy plant foods (such as added sugars, salt, refined grains, and excessive amounts of alcohol). There was no recommendation to necessarily consume smaller volumes of food or to adhere to a certain macronutrient ratio. Dietary and other lifestyle recommendations were summed up on an illustrated and laminated information sheet which was given to the participants (which has been published^22^). Apart from diet, the lifestyle recommendations were to engage in regular physical activity (≥30 min/day) and to be more mindful of taking time out to relax and of spending more time with supportive people (e.g., friends and family).

### 2.4 Assessment of parameters

All assessments and blood sampling were performed in the morning (6:00–11:00 AM) after an overnight fast. Laboratory assays have been published previously,^10^ except for those for high‐sensitivity C‐reactive protein (hs‐CRP; analyzed in serum; spectrometry: immunonephelometry; Siemens BN 2) and oxidized low‐density lipoprotein (LDL) particles (analyzed in EDTA plasma; colorimetric: human oxidized LDL ELISA, Novus Biologicals; with a Grifols Diagnostic Triturus). For hs‐CRP, participants with an infection or common cold (self‐reported at either measurement time point) were excluded from the analyses if at the time point with an infection hs‐CRP was above optimal (≥0.8 mg/L^23^, ^24^). Calculated LDL cholesterol (LDL‐C) was calculated with the Friedewald formula. Remnant cholesterol was calculated as total cholesterol minus measured LDL‐C minus high‐density lipoprotein (HDL) cholesterol (HDL‐C).^25^, ^26^ As a sensitivity analysis, remnant cholesterol was also calculated based on calculated LDL‐C (i.e., using calculated instead of measured LDL‐C). Non‐HDL cholesterol (non‐HDL‐C) was calculated as total cholesterol minus HDL‐C. Waist circumference was not assessed at 16 months (due to the COVID‐19 protocol), and oxidized LDL particles were only assessed at baseline and 10 weeks (due to the high cost). Semi‐quantitative 3‐day food protocols (based on portions of different food groups) were used to assess dietary intake. Food intake was not be assessed at 16 months as the use of the printed 3‐day food diary was not permitted due to the COVID‐19 protocol at the time and compliance with digital means of dietary assessment was expected to be extremely low in our study population. Adherence to dietary recommendations and dietary quality were assessed with the diet scores PDI, hPDI, and uPDI^12^ (the calculation of these scores has been described previously^10^). The diet scores PDI, hPDI, and uPDI are based on healthy plant food groups (whole grains, fruits, vegetables, nuts, legumes, vegetable oils, and tea and coffee), less healthy plant food groups (fruit juices, refined grains, potatoes, sugar‐sweetened beverages, and sweets and desserts) as well as animal‐source food groups (animal fat, dairy, egg, fish or seafood, meat, and miscellaneous animal‐based foods).^12^ In addition, a post hoc analysis was conducted with a modified hPDI (hPDImod), which was equivalent to hPDI except that the food groups potatoes, fish or seafood, eggs, and dairy were excluded. The rationale for this was that, although hPDI counts these food groups as “negatives” (unhealthful), potential adverse cardiovascular effects of potatoes,^27^, ^28^, ^29^, ^30^ fish,^31^ eggs,^32^, ^33^, ^34^, ^35^, ^36^ and dairy^29^, ^37^, ^38^ (compared to plant‐based protein sources) are uncertain^39^. In the context of this intervention, increases in PDI, hPDI, and hPDImod as well as a decrease in uPDI are considered desirable. Socio‐demographic parameters were assessed using questionnaires.

### 2.5 Statistical analyses

The sample size was based on a previous study (HLCP‐2 study^10^). To evaluate within‐group changes, paired *t*‐test was used for normally distributed and Wilcoxon test for non‐normally distributed data (two‐sided tests). Shapiro‐Wilk test was used to assess the data for non‐normality, and *p* < 0.05 was defined as describing a non‐normal distribution. Holm‐Bonferroni correction was conducted to adjust for multiple comparisons. Bivariate correlations were assessed with Spearman's rho correlations (two‐sided). Analyses were based on unimputed data (complete case analysis). Statistical significance was consistently set at the 0.05 level. All analyses were conducted using IBM SPSS Statistics (Version 27.0; Armonk, NY). Changes are reported as means and 95% confidence intervals.

### 2.6 Ethics statement

All participants provided written informed consent before participating in the study. The study was conducted in accordance with the Declaration of Helsinki. The study protocol was approved by the ethics committee of the Medical Association of Westphalia‐Lippe and of the University of Münster (Münster, Germany; reference: 2019‐142‐f‐S; approved 12 March 2019). The trial was registered in the German Clinical Trials Register (reference: DRKS00018846; www.drks.de).

## 3 RESULTS

### 3.1 Baseline characteristics

The flow of participants through the study is shown in Figure 1. These participants were included in the analyses (body weight; complete case analysis). Sociodemographic characteristics, smoker status, and the prevalence of overweight and obesity are shown in Table 1. Baseline values of risk markers are shown in Table 2.

**TABLE 1:** Baseline characteristics of evaluable participants (CCA; *n* = 85)

Women, *n* (%)		59 (69.4)
Age at baseline, years		58.7 ± 8.1
Overweight, *n* (%)		48 (56.5)
Obesity, *n* (%)		17 (20.0)
Smoker status, *n* (%)	Never	45 (52.9)
	Ex	33 (38.8)
	Smoker	6 (7.1)
	Missing data	1 (1.2)
Marital status, *n* (%)	Married	73 (85.9)
	Partner (unmarried)	2 (2.4)
	Single (not widowed)	5 (5.9)
	Single (widowed)	4 (4.7)
	Missing data	1 (1.2)
Educational level, *n* (%)	Lower secondary school	9 (10.6)
	Secondary school	29 (34.1)
	University entrance qualification	20 (23.5)
	University degree	26 (30.6)
	Missing data	1 (1.2)

*Note*: Age is given as mean ± standard deviation.

Abbreviation: CCA, complete case analysis.

**TABLE 2:** 16‐month analysis: Baseline and follow‐up measurements in evaluable participants (CCA)

Parameters	*n*	Baseline	10 weeks	6 months	16 months	Δ (baseline, 16 months)	*p*‐value^a^
Body weight, kg	85	80.0 ± 14.6	77.5 ± 14.2	77.2 ± 14.1	78.3 ± 14.3	−1.8 (−2.6, −1.0)	**<0.001** ^b^
BMI, kg/m^2^	85	26.7 ± 4.4	25.9 ± 4.3	25.8 ± 4.2	26.1 ± 4.4	−0.6 (−0.8, −0.3)	**<0.001** ^c^
Total cholesterol, mg/dl	80	207 ± 40	191 ± 33	199 ± 35	206 ± 35	−1 (−6, 4)	0.424^c^
LDL‐C meas., mg/dl	80	140 ± 36	129 ± 32	127 ± 29	128 ± 29	−12 (−17, −8)	<**0.001** ^c^
LDL‐C calc., mg/dl	79	124 ± 34	109 ± 29	117 ± 29	122 ± 29	−2 (−7, 2)	0.240^c^
HDL‐C, mg/dl	80	63 ± 18	62 ± 17	62 ± 16	65 ± 17	1 (−0, 3)	0.084^c^
Non‐HDL‐C, mg/dl	80	144 ± 37	128 ± 32	137 ± 32	141 ± 32	−2 (−7, 2)	0.248^c^
REM‐C, mg/dl	80	3 ± 10	0 ± 11	10 ± 8	13 ± 10	10 (8, 12)	<**0.001** ^c^
REM‐C based on LDL‐C calc., mg/dl	79	20 ± 9	18 ± 8	20 ± 8	19 ± 7	−1 (−2, 1)	0.817^c^
TAG, mg/dl	80	100 ± 47	95 ± 46	101 ± 42	99 ± 54	0 (−10, 9)	0.973^c^
Glucose, mg/dl	80	102 ± 18	100 ± 15	97 ± 11	101 ± 13	−1 (−4, 2)	0.632^c^
HbA1c, %	80	5.4 ± 0.6	5.6 ± 0.5	5.3 ± 0.4	5.5 ± 0.4	0.1 (0.0, 0.2)	**<0.001** ^c^
Insulin, μU/ml	80	10 ± 7	9 ± 6	10 ± 7	10 ± 7	0 (−1, 1)	0.586^c^
Hs‐CRP, mg/L (excl. Inf.)	59	1.4 ± 1.7	1.5 ± 2.3	1.3 ± 1.7	2.3 ± 5.5	0.9 (−1.5, 2.2)	0.222^c^
Systolic BP, mmHg	73	127 ± 14	117 ± 13	123 ± 16	127 ± 15	−1 (−4, 2)	0.642^c^
Diastolic BP, mmHg	73	78 ± 9	73 ± 8	75 ± 8	77 ± 9	−1 (−3, 1)	0.417^b^
PP, mmHg	73	49 ± 11	45 ± 10	47 ± 12	49 ± 12	0 (−3, 3)	0.949^b^
RHR, beats/min	73	66 ± 9	67 ± 11	65 ± 10	64 ± 9	−2 (−4, 0)	0.062^c^

*Note*: Values are means ± SEM. Changes are expressed as means and 95% CI. Bolded values indicates the values that are less than 0.05.

Abbreviations: BMI, body mass index; BP, blood pressure; CCA, complete case analysis; CI, confidence interval; HDL, high‐density lipoprotein; HDL‐C, HDL cholesterol; hs‐CRP, high‐sensitivity C‐reactive protein; LDL, low‐density lipoprotein; LDL‐C calc., calculated LDL‐C; LDL‐C meas., measured LDL cholesterol; non‐HDL‐C, non‐HDL cholesterol; PP, pulse pressure; REM‐C, remnant cholesterol; RHR, resting heart rate; SD, standard deviation; TAG, triglycerides.

^a^ *p*‐value for within‐group comparisons by:

^b^ Paired *t*‐test (two‐sided).

^c^ Wilcoxon test (two‐sided).

### 3.2 Changes in risk markers from baseline to 10 weeks (intensive phase)

Significant decreases were observed for body weight (−3%), BMI (−3%), waist circumference (−3%), total cholesterol (−8%), measured LDL‐C (−9%), calculated LDL‐C (−12%), oxidized LDL particles (−24%), non‐HDL‐C (−10%), remnant cholesterol (when based on measured LDL‐C [−133%] but not when based on calculated LDL‐C), glucose (−3%), insulin (−10%), systolic (−8%) and diastolic blood pressure (−6%) as well as pulse pressure (−10%). However, a small increase in HbA1c (+2%) was observed. Results remained significant after Holm‐Bonferroni correction, except for the results for glucose and insulin which became non‐significant. No other significant changes were observed (Table S1).

### 3.3 Changes in risk markers from baseline to 6 months

Significant decreases were observed for body weight (−4%), BMI (−3%), waist circumference (−4%), total cholesterol (−4%), measured LDL‐C (−10%), calculated LDL‐C (−6%), non‐HDL‐C (−5%), glucose (−5%), HbA1c (−4%) as well as systolic (−4%) and diastolic blood pressure (−4%). Remnant cholesterol significantly increased (when based on measured LDL‐C [+233%] but not when based on calculated LDL‐C), with no other significant changes. Results remained significant after Holm‐Bonferroni correction, except for the results for systolic blood pressure which became non‐significant (Table S2).

### 3.4 Changes in risk markers from baseline to 16 months

Significant decreases were observed for body weight (−2%), BMI (−2%), and measured LDL‐C (−9%; Table 2), while a small increase in HbA1c (+2%) as well as an increase in remnant cholesterol (when based on measured LDL‐C [+333%] but not when based on calculated LDL‐C) were observed, with no other significant changes (Table 2). All results remained significant after Holm‐Bonferroni correction.

### 3.5 Dietary changes from baseline to 10 weeks (intensive phase)

PDI increased by 15 points (equivalent to 5.1 [3.8, 6.5] food portions/day), hPDI increased by 32 points (equivalent to 10.8 [9.1, 12.5] food portions/day), and hPDImod increased by 26 points (equivalent to 8.6 [7.1, 10.1] food portions/day), while for uPDI a decrease of −12 points (equivalent to −3.9 [−5.3, −2.4] food portions/day) was observed (all: *p* < 0.001; *n* = 85).

### 3.6 Dietary changes from baseline to 6 months

The improvements in dietary quality from baseline to 6 months were still significant but smaller than those from baseline to 10 weeks: There were significant increases in PDI (13 points; 4.3 [2.9, 5.7] food portions/day), hPDI (24 points; 7.8 [6.1, 9.6] portions/day), and hPDImod (19 points; 6.2 [4.7, 7.8] food portions/day), while for uPDI a decrease of −7 points (−2.5 [−3.9, −1.0] food portions/day) was observed (all: *p* < 0.001; *n* = 85).

### 3.7 Bivariate correlations between diet score changes and risk marker changes

From baseline to 10 weeks, significant inverse correlations were observed for changes in hPDI and hPDImod with changes in body weight and BMI (all: *p* ≤ 0.001; Table 3).

**TABLE 3:** Correlations of 10‐week changes in PDI, hPDI, uPDI, and hPDImod with other markers

Parameter changes	PDI change		hPDI change		uPDI change		hPDImod change		*n*
	*r*	*p*‐value	*r*	*p*‐value	*r*	*p*‐value	*r*	*p*‐value	
Body weight	−0.279	0.010§	−0.360	**<0.001**	0.178	0.103	−0.342	**0.001**	85
BMI	−0.289	0.007§	−0.389	**<0.001**	0.197	0.071	−0.372	**<0.001**	85
WC	−0.063	0.568	−0.145	0.186	0.102	0.353	−0.173	0.114	85
Total cholesterol	−0.067	0.552	−0.068	0.546	0.241	0.031§	−0.214	0.057	80
LDL‐C meas.	−0.037	0.743	−0.043	0.705	0.259	0.020§	−0.203	0.071	80
LDL‐C calc.	−0.040	0.726	0.029	0.797	0.121	0.290	−0.108	0.343	79
oxLDL	−0.073	0.539	−0.097	0.412	0.099	0.404	−0.033	0.781	73
HDL‐C	0.014	0.902	−0.097	0.390	0.196	0.082	−0.148	0.189	80
Non‐HDL‐C	−0.099	0.384	−0.044	0.697	0.174	0.123	−0.189	0.093	80
REM‐C (based on LDL‐C meas.)	0.002	0.986	0.120	0.290	−0.221	0.049§	0.117	0.303	80
TAG	−0.054	0.637	−0.046	0.684	0.155	0.169	−0.158	0.161	80
Glucose	−0.072	0.523	−0.101	0.375	0.147	0.192	−0.196	0.082	80
HbA1c	0.103	0.361	−0.017	0.880	0.030	0.792	0.028	0.805	80
Insulin	0.061	0.591	−0.164	0.147	0.238	0.033§	−0.230	0.040§	80
Hs‐CRP	−0.070	0.596	−0.080	0.547	−0.012	0.927	−0.152	0.250	59
Systolic BP	0.058	0.625	0.035	0.767	−0.004	0.973	0.065	0.587	73
Diastolic BP	0.210	0.075	0.207	0.079	−0.109	0.360	0.251	0.032§	73
Pulse pressure	−0.014	0.904	−0.035	0.771	0.005	0.967	−0.019	0.871	73
RHR	−0.069	0.563	−0.145	0.220	0.021	0.862	−0.178	0.132	73

*Note*: Bolded values indicates the values that are less than 0.05.

Abbreviations: §, non‐significant after Holm‐Bonferroni correction; BMI, body mass index; BP, blood pressure; HDL, high‐density lipoprotein; HDL‐C, HDL cholesterol; hPDI, healthful PDI; hPDImod, modified hPDI; LDL, low‐density lipoprotein; LDL‐C calc., calculated LDL‐C; LDL‐C meas., measured LDL cholesterol; non‐HDL‐C, non‐HDL cholesterol; oxLDL, oxidized LDL particles; PDI, plant‐based diet index; *r*, Spearman correlation coefficient; REM‐C, remnant cholesterol; RHR, resting heart rate; TAG, triglycerides; uPDI, unhealthful PDI; WC, waist circumference.

From baseline to 6 months, significant inverse correlations were observed for changes in hPDI and hPDImod with changes in total cholesterol, LDL‐C (measured and calculated), and non‐HDL‐C (all: *p* ≤ 0.002; Table 4). Correlations of biomarker changes with changes of dietary intake at the food group level largely confirmed the associations that were observed at the diet score level (Supporting Information S1).

**TABLE 4:** Correlations of 6‐month changes in PDI, hPDI, uPDI, and hPDImod with other markers

Parameter changes	PDI change		hPDI change		uPDI change		hPDImod change		*n*
	*r*	*p*‐value	*r*	*p*‐value	*r*	*p*‐value	*r*	*p*‐value	
Body weight	−0.191	0.079	−0.265	0.014§	0.125	0.254	−0.245	0.024§	85
BMI	−0.201	0.065	−0.278	0.010§	0.129	0.240	−0.256	0.018§	85
WC	−0.056	0.608	−0.096	0.380	0.013	0.908	−0.077	0.486	85
Total cholesterol	−0.256	0.022§	−0.363	**<0.001**	0.169	0.133	−0.355	**0.001**	80
LDL‐C meas.	−0.298	0.007§	−0.343	**0.002**	0.173	0.124	−0.345	**0.002**	80
LDL‐C calc.	−0.327	0.003§	−0.397	**<0.001**	0.168	0.138	−0.396	**<0.001**	79
HDL‐C	−0.169	0.133	−0.243	0.030§	0.293	0.008§	−0.260	0.020§	80
Non‐HDL‐C	−0.237	0.034§	−0.359	**0.001**	0.119	0.293	−0.337	**0.002**	80
REM‐C (based on LDL‐C meas.)	0.115	0.308	−0.025	0.826	−0.145	0.201	0.021	0.852	80
TAG	0.128	0.257	−0.006	0.958	−0.005	0.963	0.021	0.851	80
Glucose	−0.168	0.136	−0.149	0.186	0.187	0.096	−0.211	0.060	80
HbA1c	−0.007	0.953	−0.155	0.171	0.147	0.193	−0.122	0.281	80
Insulin	0.113	0.318	−0.058	0.612	0.051	0.652	−0.055	0.629	80
Hs‐CRP	−0.026	0.844	−0.066	0.618	−0.125	0.346	−0.047	0.725	59
Systolic BP	−0.234	0.047§	−0.072	0.545	−0.022	0.853	−0.132	0.267	73
Diastolic BP	−0.190	0.107	−0.145	0.221	0.068	0.567	−0.100	0.398	73
Pulse pressure	−0.134	0.257	0.031	0.797	−0.103	0.387	−0.040	0.738	73
RHR	0.007	0.953	−0.145	0.220	−0.020	0.868	−0.076	0.521	73

*Note*: Bolded values indicates the values that are less than 0.05.

Abbreviations: §, non‐significant after Holm‐Bonferroni correction; BMI, body mass index; BP, blood pressure; HDL, high‐density lipoprotein; HDL‐C, HDL cholesterol; hPDI, healthful PDI; hPDImod, modified hPDI; LDL, low‐density lipoprotein; LDL‐C calc., calculated LDL‐C; LDL‐C meas., measured LDL cholesterol; non‐HDL‐C, non‐HDL cholesterol; PDI, plant‐based diet index; *r*, Spearman correlation coefficient; REM‐C, remnant cholesterol; RHR, resting heart rate; TAG, triglycerides; uPDI, unhealthful PDI; WC, waist circumference.

## 4 DISCUSSION

The present study had the aim of assessing potential effects of the HLCP‐3 intervention on body weight and other CVD risk markers in a sample of mostly middle‐aged and elderly individuals in rural northwest Germany (most of whom were clinically healthy). Furthermore, the study had the aim of assessing potential correlations between changes in dietary scores and risk markers. During the intensive phase of the intervention (baseline to 10 weeks), the majority of parameters significantly improved, including body weight, BMI, waist circumference, total cholesterol, measured and calculated LDL‐C, oxidized LDL particles, non‐HDL‐C, and remnant cholesterol (only when based on measured LDL‐C). While most of these parameters were still significantly decreased at 6 months, this was not the case for remnant cholesterol (based on measured LDL‐C), for which contrary to the significant decrease of −4 mg/dl from baseline to 10 weeks, a significant increase (+7 mg/dl) was observed from baseline to 6 months. This increase was even clearer when looking at changes from baseline to 16 months (+10 mg/dl; Table 2). This unexpected finding is in contrast to the significant 1‐year remnant cholesterol decrease (−3 mg/dl) which was observed in a previous study, conducted by the same research group 1 year earlier, with a nearly identical intervention program (HLCP‐2 study).^10^

The present study showed that lower intakes of sweets and desserts (Supporting Information S1), a lower uPDI as well as higher hPDI and hPDImod scores (albeit non‐significant after correction for multiple testing; Table 4) were associated with lower HDL‐C. This confirms the observation in the previous study with a nearly identical lifestyle program (HLCP‐2)^10^ as well as results from the literature that some plant‐based diets^40^ as well as higher intakes of alpha‐linolenic acid^41^ and of whole grains^42^ appear to be associated with small decreases in HDL‐C. However, the clinical relevance of this is uncertain^43^ as current evidence indicates that the quantity of HDL‐C seems to be less important as a determinant of CVD risk than the function of HDL particles.^44^

The present study shows that most of the parameters assessed were improved at 10 weeks and 6 months but that at 16 months these improvements could only be shown for body weight, BMI, and measured LDL‐C. This failure to maintain achieved improvements may have been due to decreasing compliance over time. While results from the present study show that adherence to the dietary recommendations given was similar compared to the previous study (HLCP‐2),^10^ dietary data was not available for the 16 month time point.

A strength of the present study is the assessment of a variety of CVD risk markers and multiple measurement time points. While multiple assessments increase the risk of significant findings, corrections for multiple testing were made. A considerable limitation of the present study is that it was an uncontrolled trial. Thus, causality cannot easily be inferred. Events unrelated to the intervention, such as seasonal factors,^45^, ^46^ may have influenced the results. Furthermore, the number of participants was relatively low, with a relatively large dropout rate (∼26%; Figure 1), and as the present study is an uncontrolled trial, the possibility should be taken into account that the observed improvements were not due to dietary/lifestyle changes but due to the simple fact that participants were being weighed and measured. However, the observed correlations between improved dietary quality and improved risk markers as well as the similar results observed in the previous (controlled) trial with a nearly identical lifestyle program (HLCP‐2)^10^ indicate that the observed changes appear to have at least partly been a consequence of the intervention.

The COVID‐19 pandemic may also have influenced the results of the 16‐month time point, although the number of new COVID‐19 cases in Germany appears to have been relatively low at the time (July 2020).^47^ Dietary intake was assessed with a 3‐day dietary record which had been tested in the previous study (HLCP‐2^10^). However, the dietary questionnaires were not validated, and some misreporting of food intake is possible. In order to minimize this imprecision, dietary evaluation in the present study was based on food scores (PDI, hPDI, and uPDI) rather than individual foods.

## 5 CONCLUSIONS

The present study (HLCP‐3) was able to replicate the findings of a previous study (HLCP‐2) in terms of significant improvements in dietary behavior, body weight, BMI, and waist circumference (but not resting heart rate or remnant cholesterol) at the end of the study.^10^ Increases in hPDI correlated with some improved risk markers. The results appear to confirm a large body of evidence that the recommendation of a healthy plant‐based dietary pattern is an effective and actionable public health tool for long‐term body weight control. The results, however, indicate that the lifestyle program requires further optimization in order to achieve stronger long‐term improvements in other cardiovascular risk markers. The present study contributes the finding that a healthy lifestyle program including the recommendation to follow a healthy plant‐based diet can be an acceptable and feasible intervention for a population of middle‐aged and elderly individuals in rural Germany. In addition, the present study contributes the novel finding that changes in the dietary scores PDI, hPDI, and uPDI as well as the novel hPDImod may be suitable parameters for intervention trials.

## AUTHOR CONTRIBUTIONS

**Christian Koeder**: Conceptualization; Methodology; Validation; Formal analysis; Investigation; Data curation; Writing – original draft; Writing – review & editing; Project administration. **Dima Alzughayyar**: Conceptualization; Methodology; Investigation; Data curation; Writing – review & editing. **Corinna Anand**: Conceptualization; Methodology; Investigation; Data curation; Writing – review & editing; Project administration. **Ragna‐Marie Kranz**: Conceptualization; Methodology; Investigation; Data curation; Writing – review & editing; Project administration. **Sarah Husain**: Conceptualization; Methodology; Investigation; Data curation; Writing – review & editing; Project administration. **Nora Schoch**: Conceptualization; Methodology; Investigation; Data curation; Writing – review & editing; Project administration. **Andreas Hahn**: Methodology; Formal analysis; Writing – original draft; Writing – review & editing; Supervision; **Heike Englert**: Conceptualization; Methodology; Investigation; Writing – original draft; Writing – review & editing; Project administration; Supervision; Funding acquisition. All authors read and approved the final manuscript.

## CONFLICT OF INTEREST

We have no conflict of interest to disclose.

## Supporting information

Supporting Information S1
