# Supplementary material for: The healthful plant‐based diet index as a tool for obesity prevention—The healthy lifestyle community program cohort 3 study
Source: Obes Sci Pract. 2022 Dec 25;9(3):296–304. doi: 10.1002/osp4.649 (PMC10242251; doi:10.1002/osp4.649)
Supplement: Supplementary file 1 — Supporting Information S1 [file OSP4-9-296-s001.docx]

**Supplementary material**

**The healthful plant-based diet index as a tool for obesity prevention – the Healthy Lifestyle Community Program cohort 3 study**

**Authors**

Christian Koeder, Dima Alzughayyar, Corinna Anand, Ragna-Marie Kranz, Sarah Husain, Nora Schoch, Andreas Hahn, Heike Englert

# **Corresponding author**

Christian Koeder, [koeder@fh-muenster.de](mailto:koeder@fh-muenster.de)

# **Bivariate correlations between changes in food intake (food group level) and risk marker changes**

Correlations of biomarker changes with changes of dietary intake at the food group level largely confirmed the associations that were observed at the diet score level. The following correlations with correlation coefficients of r ≥0.3 and p ≤0.001 were observed: from baseline to 10 weeks, changes in legume intake inversely correlated with changes in body weight (r = -0.346) and BMI (r = -0.351; n = 85), while changes in the intake of sweets and desserts positively correlated with changes in total cholesterol (r = 0.399) and HDL-C (r = 0.371). TAG changes inversely correlated with changes in vegetable intake (r= -0.398; n = 80; this was also the case for remnant cholesterol based on calculated LDL-C; r = -0.383; n = 79). From baseline to 6 months, changes in nut intake inversely correlated with changes in systolic blood pressure (r = -0.394). No other highly significant (p ≤0.001) correlations at the food group level were observed.

**Table S1**

| **Table S1.** *10-Week Analysis: Baseline and Follow-Up Measurements (CCA)* | | | | | |
| --- | --- | --- | --- | --- | --- |
| **Parameters** | **n** | **Baseline** | **10 weeks** | Δ**(baseline, 10 weeks)** | **p-value** § |
| Body weight, kg | 85 | 80.0 ± 14.6 | 77.5 ± 14.2 | -2.6 (-3.0, -2.1) | **<0.001** a |
| BMI, kg/m^2^ | 85 | 26.7 ± 4.4 | 25.9 ± 4.3 | -0.8 (-1.0, -0.7) | **<0.001** b |
| WC, cm | 85 | 92.2 ± 14.0 | 89.3 ± 13.4 | -2.9 (-3.5, -2.3) | **<0.001** b |
| Total cholesterol, mg/dl | 80 | 207 ± 40 | 191 ± 33 | -17 (-22, -12) | **<0.001** b |
| LDL-C meas., mg/dl | 80 | 140 ± 36 | 129 ± 32 | -12 (-16, -7) | <**0.001** b |
| LDL-C calc., mg/dl | 79 | 124 ± 34 | 109 ± 29 | -15 (-19, -11) | **<0.001** b |
| oxLDL, pg/ml | 73 | 1481 ± 415 | 1124 ± 421 | -358 (-463, -252) | <**0.001** b |
| HDL-C, mg/dl | 80 | 63 ± 18 | 62 ± 17 | -1 (-3, 1) | 0.120 b |
| non-HDL-C, mg/dl | 80 | 144 ± 37 | 128 ± 32 | -15 (-19, -11) | **<0.001** b |
| REM-C, mg/dl | 80 | 3 ± 10 | 0 ± 11 | -4 (-5, -2) | **<0.001** b |
| REM-C based on LDL-C calc., mg/dl | 79 | 20 ± 9 | 18 ± 8 | -1 (-3, 0) | 0.087 b |
| TAG, mg/dl | 80 | 100 ± 47 | 95 ± 46 | -5 (-12, 3) | 0.136 b |
| Glucose, mg/dl | 80 | 102 ± 18 | 100 ± 15 | -3 (-5, 0) | 0.014 b $ |
| HbA1c, % | 80 | 5.4 ± 0.6 | 5.6 ± 0.5 | 0.1 (0.1, 0.2) | **<0.001** b |
| Insulin, µU/ml | 80 | 10 ± 7 | 9 ± 6 | -1 (-2, 0) | 0.019 b $ |
| hs-CRP, mg/l | 59 | 1.4 ± 1.7 | 1.5 ± 2.3 | 0.1 (-0.4, 0.6) | 0.142 b |
| Systolic BP, mmHg | 73 | 127 ± 14 | 117 ± 13 | -10 (-13, -7) | **<0.001** b |
| Diastolic BP, mmHg | 73 | 78 ± 9 | 73 ± 8 | -5 (-7, -4) | **<0.001** a |
| Pulse pressure, mmHg | 73 | 49 ± 11 | 45 ± 10 | -5 (-7, -2) | **<0.001** a |
| RHR, beats/min | 73 | 66 ± 9 | 67 ± 11 | 1 (-1, 3) | 0.354 b |
| Values are means ± SEM. Changes are expressed as means and 95% CI; CCA: complete case analysis; SD: standard deviation; CI: confidence interval; BMI: body mass index; WC: waist circumference; LDL-C meas.: measured LDL cholesterol; LDL-C calc.: calculated LDL-C; oxLDL: oxidized LDL particles; non-HDL-C: non-HDL cholesterol; REM-C: remnant cholesterol; HDL-C: HDL cholesterol; TAG: triglycerides; hs-CRP: high-sensitivity C-reactive protein; BP: blood pressure; RHR: resting heart rate;  § p-value for within-group comparisons of changes (baseline to 10 weeks) by:  ^a^ paired t-test (two-sided)  ^b^ Wilcoxon test (two-sided); $: non-significant after Holm-Bonferroni correction | | | | | |

**Table S2**

| **Table S2.** *6-Month Analysis: Baseline and Follow-Up Measurements (CCA)* | | | | | |
| --- | --- | --- | --- | --- | --- |
| **Parameters** | **n** | **Baseline** | **6 months** | Δ**(baseline, 6 months)** | **p-value** § |
| Body weight, kg | 85 | 80.0 ± 14.6 | 77.2 ± 14.1 | -2.8 (-3.5, -2.1) | **<0.001** a |
| BMI, kg/m^2^ | 85 | 26.7 ± 4.4 | 25.8 ± 4.2 | -0.9 (-1.2, -0.7) | **<0.001** b |
| WC, cm | 85 | 92.2 ± 14.0 | 88.8 ± 12.9 | -3.4 (-4.3, -2.5) | **<0.001** b |
| Total cholesterol, mg/dl | 80 | 207 ± 40 | 199 ± 35 | -9 (-14, -3) | **0.002** a |
| LDL-C meas., mg/dl | 80 | 140 ± 36 | 127 ± 29 | -14 (-19, -9) | <**0.001** b |
| LDL-C calc., mg/dl | 79 | 124 ± 34 | 117 ± 29 | -7 (-11, -3) | **0.003** a |
| HDL-C, mg/dl | 80 | 63 ± 18 | 62 ± 16 | -2 (-4, 0) | 0.060 b |
| non-HDL-C, mg/dl | 80 | 144 ± 37 | 137 ± 32 | -7 (-11, -2) | **0.005** b |
| REM-C, mg/dl | 80 | 3 ± 10 | 10 ± 8 | 7 (6, 9) | <**0.001** b |
| REM-C based on LDL-C calc., mg/dl | 79 | 20 ± 9 | 20 ± 8 | 0 (-2, 2) | 0.386 b |
| TAG, mg/dl | 80 | 100 ± 47 | 101 ± 42 | 1 (-7, 8) | 0.313 b |
| Glucose, mg/dl | 80 | 102 ± 18 | 97 ± 11 | -5 (-8, -2) | <**0.001** b |
| HbA1c, % | 80 | 5.4 ± 0.6 | 5.3 ± 0.4 | -0.2 (-0.2, -0.1) | <**0.001** b |
| Insulin, µU/ml | 80 | 10 ± 7 | 10 ± 7 | 0 (-1, 1) | 0.288 b |
| hs-CRP, mg/l | 59 | 1.4 ± 1.7 | 1.3 ± 1.7 | -0.1 (-0.4, 0.2) | 0.861 b |
| Systolic BP, mmHg | 73 | 127 ± 14 | 123 ± 16 | -5 (-8, -1) | 0.008 b $ |
| Diastolic BP, mmHg | 73 | 78 ± 9 | 75 ± 8 | -3 (-4, -1) | **0.002** a |
| Pulse pressure, mmHg | 73 | 49 ± 11 | 47 ± 12 | -2 (-5, 1) | 0.161 a |
| RHR, beats/min | 73 | 66 ± 9 | 65 ± 10 | -1 (-3, 1) | 0.230 b |
| Values are means ± SEM. Changes are expressed as means and 95% CI; CCA: complete case analysis; SD: standard deviation; CI: confidence interval; BMI: body mass index; WC: waist circumference; LDL-C meas.: measured LDL cholesterol; LDL-C calc.: calculated LDL-C; non-HDL-C: non-HDL cholesterol; REM-C: remnant cholesterol; HDL-C: HDL cholesterol; TAG: triglycerides; hs-CRP: high-sensitivity C-reactive protein; BP: blood pressure; RHR: resting heart rate;  § p-value for within-group comparisons of changes (baseline to 6 months) by:  ^a^ paired t-test (two-sided)  ^b^ Wilcoxon test (two-sided); $: non-significant after Holm-Bonferroni correction | | | | | |
